# Curare and GenExVis: a versatile toolkit for analyzing and visualizing RNA-Seq data

**DOI:** 10.1186/s12859-024-05761-2

**Published:** 2024-03-29

**Authors:** Patrick Blumenkamp, Max Pfister, Sonja Diedrich, Karina Brinkrolf, Sebastian Jaenicke, Alexander Goesmann

**Affiliations:** Bioinformatics and Systems Biology, Justus Liebig University Giessen, 35392 Giessen, Germany

**Keywords:** RNA-Seq, Differential gene expression, Data visualization, FAIR, Reproducibility

## Abstract

Even though high-throughput transcriptome sequencing is routinely performed in many laboratories, computational analysis of such data remains a cumbersome process often executed manually, hence error-prone and lacking reproducibility. For corresponding data processing, we introduce Curare, an easy-to-use yet versatile workflow builder for analyzing high-throughput RNA-Seq data focusing on differential gene expression experiments. Data analysis with Curare is customizable and subdivided into preprocessing, quality control, mapping, and downstream analysis stages, providing multiple options for each step while ensuring the reproducibility of the workflow. For a fast and straightforward exploration and visualization of differential gene expression results, we provide the gene expression visualizer software GenExVis. GenExVis can create various charts and tables from simple gene expression tables and DESeq2 results without the requirement to upload data or install software packages. In combination, Curare and GenExVis provide a comprehensive software environment that supports the entire data analysis process, from the initial handling of raw RNA-Seq data to the final DGE analyses and result visualizations, thereby significantly easing data processing and subsequent interpretation.

**Supplementary Information:**

The online version contains supplementary material available at 10.1186/s12859-024-05761-2.

## Background

### RNA-Seq and differential gene expression

RNA sequencing (RNA-Seq) is a universal tool for analyzing transcriptomes of an organism. It has mostly replaced the formerly used microarrays in many fields due to its potential to gain an exhaustive overview of the transcriptional landscape. In this process, the RNA molecules are reverse-transcribed into cDNAs, which are then sequenced on contemporary high-throughput instruments. After an initial quality assessment, these sequence fragments are aligned to the corresponding reference genome, and based on its annotation, the respective gene is identified, and its expression quantified [1, 2]. One of the most common applications of RNA-Seq is the quantification and comparison of transcriptomic features (e.g., messenger RNAs (mRNAs)), known as differential gene expression (DGE) analysis. These RNA-Seq expression studies are designed to compare two or more conditions, e.g., wild type vs mutant. The comparison between conditions is performed after correcting for various factors such as sequencing depth variations or gene length bias, and subsequently, changes in gene expression levels can be deduced. Since RNA-Seq does not allow for inferring gene expression quantitatively, DGE analyses are typically performed based on fold changes between two conditions [2, 3].

A myriad of bioinformatics tools is nowadays available for the processing of RNA-Seq data, making it difficult for researchers to come up with a reasonable combination to be used in conjunction with their data. Common choices include various tools that might be employed for sequence preprocessing and quality control (QC) (e.g., FastQC [4], Trim Galore [5], or fastp [6]). At the same time, selecting the most suitable reference alignment algorithm mostly depends on the characteristics of the studied organism. Commonly used tools for viruses and prokaryotes are Bowtie 2 [7] or BWA [8], while for eukaryotes, a splicing-aware aligner such as STAR [9] is typically employed. For statistical evaluation, several packages for the R statistical environment are available, most prominently the edgeR [10], limma [11], and DESeq2 [12] algorithms, with the latter as the most commonly used package. edgeR and DESeq2, both written explicitly for RNA-Seq, use generalized linear models based on negative binomial distribution and differ in how read counts are normalized internally. On the other hand, limma uses linear models and was written with Microarray datasets in mind but also supports RNA-Seq data. Since no attempt fully captures the biological reality, the optimal approach depends on both the study design and the organism’s properties, and researchers might want to apply and evaluate different approaches for an optimal outcome.

### Automated differential gene expression analysis

In the field of high-throughput data analysis, workflow systems are becoming increasingly important and are also indispensable to ensure the reproducibility of results. They easily enable the chaining of sub-steps into a linear flow and provide scalability by parallelizing execution on modern high-performance computing (HPC) clusters or cloud infrastructures. Since many workflow systems also support containers (e.g., Docker) and virtual environments (e.g., conda [13]), results of workflow runs lead to reproducible and comprehensible results that comply with FAIR data principles [14]. Nextflow [15] and Snakemake [16] are the most widely used workflow systems in bioinformatics, and initiatives such as nf-core [17] offer community-maintained workflows. However, using and adapting them to one’s data to achieve the best possible result requires expertise that the user often does not possess. Currently, multiple approaches exist for automatizing RNA-Seq analyses. These can be divided into automated and toolkit workflow solutions (Additional file 3: Table S1). Tools with automated workflows like Viper [18], R-peridot [19], TRAPLINE[20], nf-core/rnaseq + nf-core/differentialabundance [21, 22], or hppRNA [23] provide pre-built pipelines which perform parts of or all steps from data preprocessing over read alignment to subsequent statistical analysis and visualization of results. These solutions offer fast and easy-to-use workflows for most RNA-Seq analyses but often lack customizability, either by using fixed tools or not allowing parameter changes. This can lead to pipelines restricted to specific datasets instead of offering general RNA-Seq solutions. In contrast, platforms like Galaxy [24] provide access to various applications for RNA-Seq analysis. This enables users to build their workflows by chaining different tools. However, users are required to upload potentially sensitive data to a publicly available Galaxy server or host and configure a local personal Galaxy instance. The latter requires knowledge about hosting a server and installing tools in Galaxy. Also, the wide range of tool options without any guidelines requires users to possess substantial bioinformatics expertise to identify reasonable tool combinations.

### Visualization

As it becomes more accessible and more affordable to conduct DGE experiments, the number of available datasets has also increased significantly, thereby raising new challenges for the exploration and interpretation of analysis results. The larger the number of datasets of an individual experiment, the more difficult it gets to keep track of them and to interpret respective results further. Different tools have been developed to support scientists with these tasks (Additional file 3: Table S2). These tools use the results of DGE analysis methods like DESeq2 to create various charts, tables, and reports of all relevant findings. One way to visualize such data is to use R packages like ViDGER [25] and DGEReport [26], featuring many R functions to create customized charts and help users to find interesting data points. These packages are quite powerful and versatile; however, they require advanced knowledge of the R programming environment and are, therefore, only suitable for some users. Alternatively, tools like DrEdGE [27], DEBrowser [28], PIVOT [29], and Degust [30] start a web server where users can import their data and navigate through miscellaneous interactive charts and tables. Of disadvantage, however, is that these tools require either the installation of a local web server via R Shiny [31] or uploading personal or unpublished data to a public web server.

### Curare and GenExVis offer flexible yet versatile RNA-Seq data processing and interpretation

The Curare/GenExVis combination was developed to deliver a flexible workflow solution for RNA-Seq data analysis. With Curare, we developed an RNA-Seq analysis workflow builder aiming to fill the gap between easy-to-use, full-automated workflows and versatile toolkits. It addresses shortcomings of modern tools by providing a flexible, reproducible, and automatable environment for processing RNA-Seq data without creating a necessity to distribute potentially sensitive data to third parties. By integrating conda environments and detailed workflow reports, scientists are encouraged to follow FAIR data principles. After a successful Curare run, GenExVis helps scientists explore and interpret any DGE results by providing an offline usable interactive graphical user interface (GUI) for DESeq2 tables and visualizations on Windows and Linux. Without any installation, users can create various common DGE visualizations, focus on customized gene subsets, and explore their data in different tables.

## Implementation

### Curare

We developed the Customizable and Reproducible Analysis pipeline for RNA-Seq Experiments (Curare) to process high-throughput transcriptomics data. Curare is a modular application implemented in Python 3 that easily defines and executes standardized analysis workflows. Due to the internal usage of Snakemake and its power to parallelize processes, Curare is fully scalable and an ideal solution for efficiently analyzing large-scale, high-throughput data.

**Fig. 1:**
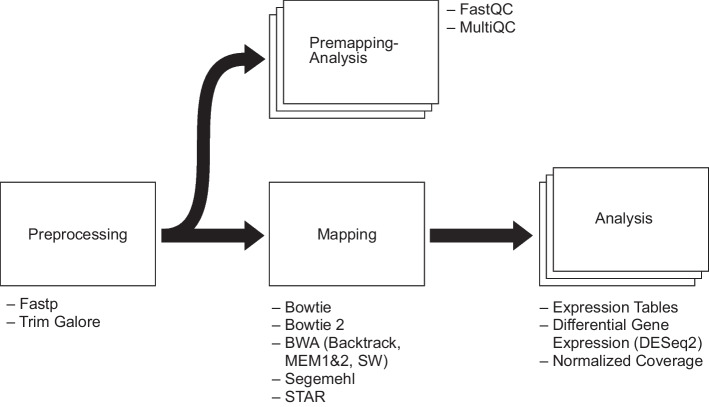
Structure of Curare and its available modules. Curare is modularized into four categories: “Preprocessing,” “Premapping-Analysis,” “Mapping,” and “Analysis.” After running a “Preprocessing” module, the sequencing reads can be analyzed by “Premapping” modules and aligned to the reference genome by “Mapping” modules. Aligned reads are afterward analyzed by modules in the “Analysis” category

Most RNA-Seq analyses are subdivided into steps for preprocessing/quality control, mapping, and analysis. Curare also follows this workflow structure by organizing its workflow sections in Preprocessing, Premapping(-Analysis), Mapping, and Analysis (Fig. 1). Each step is implemented via miscellaneous modules for the corresponding processing phase. Curare relies upon conda for the reproducibility of the analysis and automatically installs all required dependencies for selected modules. Within these standardized conda environments, the complete workflows can be executed. Each step is dynamically configured, and a researcher employing Curare can choose from different available alternatives that best match the corresponding data to be processed, e.g., the choice of a splice-aware aligner for RNA-Seq data originating from eukaryotes.

For compatibility reasons with all existing and future modules, Curare established interfaces for each module category. ‘Preprocessing’ modules must always output gzipped FASTQ files, and ‘Premapping” and ‘Mapping’ must use these FASTQ files as input. ‘Mapping’ must output BAM files, and ‘Analysis’ modules use the BAM files as input. This modularity guarantees future extensibility, as novel computational approaches or statistical methods can be included in the software without much effort.

‘Preprocessing’ comprises the first step in an RNA-Seq analysis, where the raw input sequences are processed to filter/trim low-quality reads and remove sequencing adapters. Trim Galore and fastp are two universal and fully automated solutions for adapter trimming and quality filtering included in Curare. ‘Premapping’ is a module executed after the ‘Preprocessing’ step for all analyses on preprocessed FASTQ data, e.g., quality control, remaining adapter content, or sequence length distribution. FastQC and MultiQC [32] are integrated into Curare as established tools for sequence data quality control. This enables users to quickly assess the preceding processing phase's effectiveness and adapt preprocessing/mapping settings for future runs. The ‘Mapping’ step supports all major alignment tools commonly applied for transcriptomics datasets, such as STAR or Bowtie 2. In addition, we incorporated support for tools that early users explicitly requested from the Curare application. With Bowtie 1 and 2 [7, 33], Segemehl [34], and BWA (BWA-MEM 1 and 2, Backtrack, SW) [8, 35], a variety of read-mapping tools for bacterial data are provided. Curare relies on the commonly used STAR aligner for organisms capable of splicing. For long-read sequencing, Minimap2 [36] was implemented. ‘Analysis’ is the final step of the workflow and provides multiple analysis scripts executed on the produced mapping results. Typically, DGE analyses are performed contrasting two or more different conditions to identify transcriptional changes. For this, Curare provides two DGE modules. These modules utilize featureCounts [37] for gene counting and DESeq2 or edgeR to determine differentially expressed genes. They also provide additional statistics and visualizations of gene composition, gene feature distribution, and sample similarity. If the processed dataset includes more than two conditions, the DGE module will create DESeq2/edgeR results for all possible condition combinations and summarize the results in structured spreadsheets. Besides these spreadsheets, the DGE analysis provides a normalized expression count table, vector graphics of the principal component analysis, and a correlation heatmap of the dataset. In rare circumstances where manual optimization of DESeq2/edgeR analyses might be required, it can easily be achieved by importing Curare results into the R statistical environment for additional processing. In addition, Curare generates bigWig and bedgraph files via deeptools’ bamCoverage [38], which can be used for downstream visualizations of read coverage with tools like IGV [39] or the UCSC Genome Browser [40]. In cases where a count table without any statistical analysis is needed, Curare also provides a “Count Table” module that calculates expression count tables from genome annotation and mapping results.

Before starting an actual Curare run, the interactive command line wizard collects desired analysis steps, can assist with downloading a reference genome/annotation and creates configuration templates for sample and pipeline parameters. These two templates are simple TSV and YAML files, respectively, and can be manipulated with any text editor. The sample file provides information about the sample, like name, file path, or condition, while the pipeline definition is provided as a YAML file with sections describing each module to be used in the dynamically generated workflow. The user can specify tool-specific settings (*e.g.,* Bowtie 2’s alignment mode) or provide mandatory input files (*e.g.,* a genome annotation in GFF format). All modules are pre-configured, so the default Curare parameters already provide reasonable DGE analysis settings. Only for parameters where no reasonable default can be determined or experiment-specific files (e.g., reference genome, annotation) are necessary user input is mandatory. For standard files in RNA-Seq experiments like reference genomes and genome annotations, Curare focuses on one file format each. In this case, it specializes in FASTA and GFF3, respectively. Afterward, Curare validates the existence of files, creates a customized Snakemake workflow, and executes it. During the execution of the workflow, Snakemake parallelizes individual steps. If workflow execution fails, Curare allows the inspection of log files to identify the underlying cause, and due to its modularized structure, failed workflows can be restarted without the need to re-execute steps that were previously completed successfully. Finally, Curare creates a graphical HTML report of all the steps inside the used workflow. Each module creates its own page in this report with detailed information about settings and results. In compliance with the FAIR principles, Curare also guarantees the reproducibility of all analysis steps by collecting information about all tool versions used; this information is also included in the graphical report.

### GenExVis

Despite the availability of sophisticated software tools for DGE analysis, manual exploration by an experienced scientist with in-depth field knowledge is still considered the gold standard. For this, obtaining a coarse overview of the processed data is necessary, but detailed inspection must also be supported. For this, we have developed the Gene Expression Visualizer GenExVis. GenExVis is an interactive desktop application implemented in JavaScript and NW.js [41] for exploring and visualizing bacterial DGE data created with DESeq2. It is compatible with Linux and Windows and can be used without any installation or dependencies.

GenExVis uses expression data (e.g., featureCounts results) and DESeq2 results to create interactive and highly customizable visualizations. Therefore, a tab-separated gene expression abundance table with one row per gene and the typical output of DESeq2 runs (tab-separated file with one gene per row and columns for base mean, log2 fold change, *p*-value, adjusted p-value, etc.) is required. Users are provided with a table with various filtering options after initial data import for a first result overview. Besides commonly used DGE visualizations for finding and evaluating differentially expressed genes, such as volcano plots and MA plots (log2 fold change vs. (adjusted) *p*-value or base mean, respectively), users can access various other charts. GenExVis offers multiple histograms for different DGE statistics (e.g., fold change) to provide a complete overview of the whole dataset. Users can also compare samples inside and between conditions on (normalized) gene count level, an important check to evaluate sample deviation. All these plots can be exported as publication-ready images (e.g., PNG, PDF, and SVG). For more detailed insights into specific parts of a transcriptome, it is possible to filter the expression data based on various thresholds (solo or combined), like minimal fold change or maximal *p*-value. These so-called subsets can then be used for every available visualization in GenExVis.

While GenExVis has been custom-tailored to be used with the Curare tool, it also supports handling arbitrary expression matrix data, for example, generated by the featureCounts software. Hence, it is also easily applicable to data obtained from other DGE workflows.

## Results

To illustrate the results of a typical DGE analysis conducted with Curare, we applied the following Curare workflow to a *Myxococcus xanthus* RNA-Seq study from Kuzmich et al*.* [42]. This study analyzes the stages of *M. xanthus’* biphasic life cycle on their transcript levels. With access to nutrients, *M*. *xanthus* forms predatory swarms, but in their absence, it builds spore-filled fruiting bodies. The corresponding RNA-Seq dataset of Kuzmich et al*.* (EBI ArrayExpress E-MTAB-11043; https://www.ebi.ac.uk/biostudies/arrayexpress/studies/E-MTAB-11043) is comprised of ten *M. xanthus* RNA sequencing samples originating from five different time points with two biological replicates each: non-starved cells (0 h) and 6 h, 12 h, 18 h, and 24 h under submerged conditions. We configured the Curare workflow to use the following modules: fastp for preprocessing, MultiQC (including FastQC) for quality control, Bowtie 2 as the read aligner, and DGE as well as normalized coverage as analyses (Results available at Zenodo 10.5281/zenodo.10362480). The complete and annotated genome of *M. xanthus* DK 1622 (NCBI accession NC_008095.1) was used as a reference.

The workflow begins with running fastp on every FASTQ file. Applying default settings suggested by Curare, all bases at the beginning or the end of a read with Phred scores smaller than 20 were trimmed, and reads shorter than 15 bases or with an average Phred score of less than 15 were discarded. After trimming and filtering reads, FastQC reports were created containing information about read length distribution, base qualities, GC content, and possible adapter residue. While FastQC creates reports for each FASTQ file separately, MultiQC is afterward applied to summarize all individual reports.

**Fig. 2:**
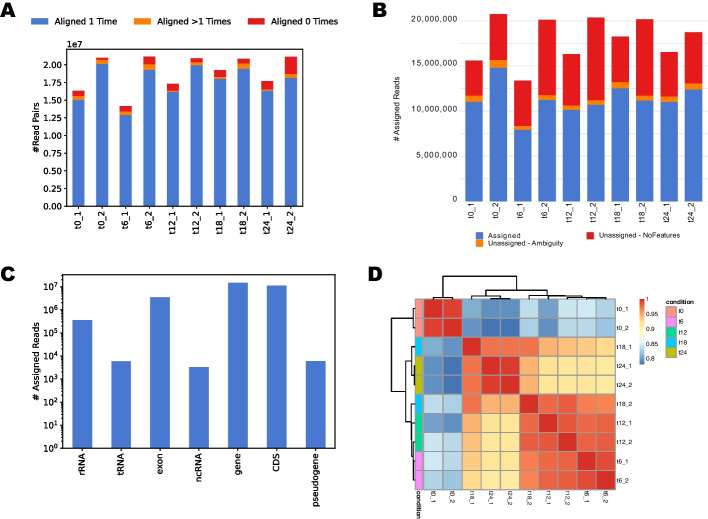
Visualization examples of Curare report. **A** Barchart shows a Curare run’s mapping results (Bowtie 2) of a Curare run. **B** Barchart shows the number of mapped reads assigned to CDS by featureCounts. **C** Barchart displays the number of mapped reads assigned to different feature types by featureCounts. **D** Correlation heatmap using Pearson correlation coefficient. All CDS with at least ten counts (normalized with DESeq2) were used for the correlation

To match the characteristics of the original study, the Curare workflow was configured to use Bowtie 2 as the read aligner. Between 88 and 98% of the reads were successfully aligned to the reference genome. The mapping statistics are summarized in XLSX files and available in the final HTML report (Fig. 2A). The aligned sample reads were saved in BAM format and indexed for downstream visualization with common genome browsers like IGV. Unmapped and discordantly aligned reads are additionally saved to separate BAM files for possible troubleshooting analyses in case of low alignment rates.

The aligned reads were subsequently allocated to individual genes with featureCounts. Here, between 50 and 70% of the aligned reads were assigned to coding sequences (CDS) in *M. xanthus* DK 1622, while the remaining reads were mapped to positions either without an annotated CDS or at ambiguous positions (Fig. 2B). Curare also provides summary reports for reads aligned to other features (*e.g.,* rRNA, tRNA, ncRNA) (Fig. 2C). This way, a user can quickly assess how well sample preparation steps such as rRNA depletion worked and what the general RNA composition looks like. A sample correlation heatmap (Fig. 2D) and a principal component analysis (PCA) are supplied for an overview of correlations between all samples, allowing visually identifying sample consistency. The actual DGE analysis was performed using DESeq2 (see Additional file 1). From 7271 annotated CDS in the *M. xanthus* DK 1622 reference genome*,* 4688 were significantly differentially expressed (*p*-value < 0.05) after 6 h. From these, 2,336 were identified as up- and 2352 as downregulated (771 and 612 with fold changes > 4, respectively). After 12/18/24 h, the number of significantly expressed genes increased/decreased to 4,873, 4,802, and 4,657, respectively (always in comparison to 0 h). Besides TSV files for every possible comparison containing the raw DESeq2 results, Curare presents all DESeq2 results in structured spreadsheets. In each sheet, all DESeq2 comparisons of one fixed condition (e.g., 0 h vs. 6 h/12 h/18 h/24 h) are collected and presented (Additional file 1).

**Fig. 3:**
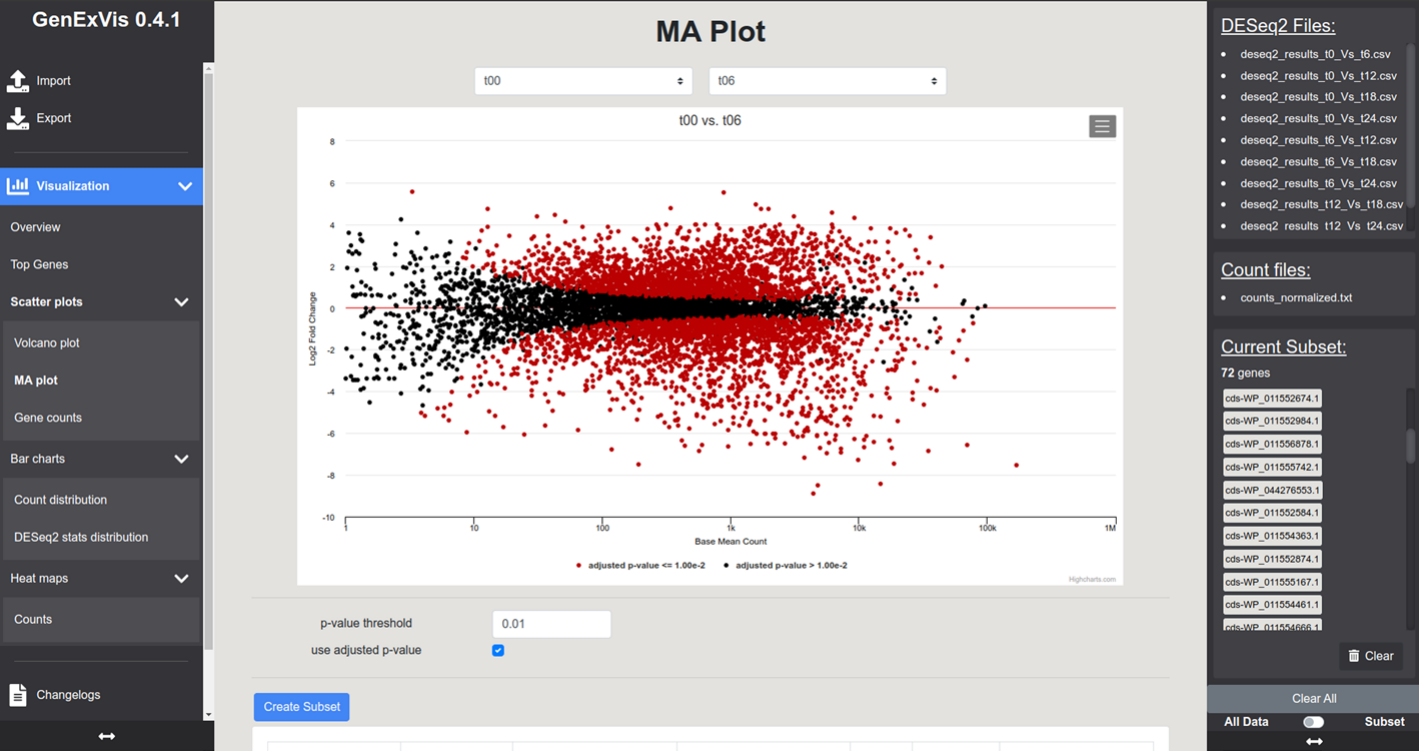
Graphical user interface of GenExVis. The interface is divided into three parts: A navigation bar on the left, the main view in the center, and a data bar on the right. The navigation bar shows options for import and export, various visualizations, and information about GenExVis. The main view changes depending on the selected visualization and can be maximized by minimizing the bars on the left and right. In this example, an MA plot can be seen. The data bar summarizes imported DESeq2 and expression count files and allows to switch between the complete dataset and a subset of features for visualizations

For further exploration, all DESeq2 result tables and the normalized expression count table created by Curare were imported to GenExVis. Plots like the volcano plot and MA plot helped to obtain an overview of expression levels between different conditions (Fig. 3 and Additional file 3: Fig. S1). Following Kuzmich et al*.*, a subset of the 72 genes of interest was created and analyzed on differentially expressed genes (Examples in Additional file 3: Fig. S1A–C). Via the Top Genes listing, the most significant and highest differentially expressed genes could be identified (Additional file 3: Fig. S1C). The results were also exported in tabular format (see Additional file 2).

## Discussion and conclusion

With Curare, we provide a comprehensive solution that significantly eases the challenges commonly encountered in the high-throughput computational analysis of RNA-Seq data. Curare enables scientists to process their datasets comfortably without requiring a thorough knowledge of bioinformatics tools or workflow systems.

Various tools are available to aid researchers with the computational analysis of such data. However, these often need more flexibility and scalability, assume users have experience in RNA-Seq analysis, or require uploading data to external servers.

In Curare, users are guided through creating their own RNA-Seq workflows by the interactive Curare wizard and well-documented descriptions next to each user input. As illustrated in our use case above, Curare’s initial setup and configuration for an experimental design can be achieved within a few minutes. After running the Curare wizard and filling in all required experiment information, no further user input is required. Curare will automatically install all the necessary tools and run the workflow.

Curare already covers common and established transcriptomics approaches; nonetheless, RNA-Seq remains a fast-moving field, and the modularity of Curare ensures that improved methods are easily integrated once they become available. Due to the usage of Snakemake and its simplicity of writing workflows, future enhancements (e.g., novel algorithms for RNA-Seq alignment or statistical assessment) of the Curare tool are easily achievable. Already used in multiple published and unpublished projects [42, 43], Curare will extend its feature sets based on user feedback and upcoming new projects. By controlling the number of parallel workflow steps, Curare is easily scaled to make the most efficient use of available hardware resources.

Curare also strives to fulfill the FAIR principles by revealing and saving the complete executed pipeline, including a list of used software with their installed versions.

GenExVis is a novel desktop application for the graphical exploration and visualization of DGE analysis results. While it perfectly complements the Curare software, GenExVis is based on established file formats and, therefore, in no way limited to results obtained with Curare.

Since GenExVis directly supports the native output files generated by DESeq2, it represents a versatile solution for the interactive exploration and interpretation of gene expression data from contemporary sequencing devices.

Compared to other visualization tools for DGE data, GenExVis does not require any installation and runs locally on every Windows and Linux computer. It can be used without any knowledge of programming languages, and it does not require hosting a server or uploading data to public websites.

Combined, the novel tools Curare and GenExVis aid researchers in processing and efficiently analyzing their RNA-Seq data, enabling them to obtain reproducible results. Adequate and meaningful charts are provided to support the convenient identification of all relevant aspects of their data, allowing rapid creation of high-quality visualizations and easing successful data interpretation.

### Supplementary Information

**Additional file 1:** Curare DGE results t0 vs. rest.

**Additional file 2:** GenExVis subset export of t0 vs t6.

**Additional file 3:** Supplementary figures and tables.

## Data Availability

Project name: Curare, Project home page: https://github.com/pblumenkamp/Curare, Operating system: Linux, Programming language: Python, JavaScript, R, License: GNU GPL v3.0, Restrictions: -Project name: GenExVis, Project home page: https://github.com/pblumenkamp/GenExVis, Operating system: Windows, Linux, Programming language: JavaScript, License: MIT, Restrictions: GenExVis comes with a non-commercial “CC BY-NC 3.0 US” license from Highcharts. RNA-Seq datasets used in the results can be found at EBI ArrayExpress E-MTAB-11043: https://www.ebi.ac.uk/biostudies/arrayexpress/studies/E-MTAB-11043, The Curare results are available at Zenodo: https://zenodo.org/records/10362480 (10.5281/zenodo.10362480).

## Abbreviations

CDS: Coding sequences
Curare: Customizable and reproducible analysis pipeline for RNA-Seq experiments
DGE: Differential gene expression
GenExVis: Gene expression visualizer
GUI: Graphical user interface
HPC: High-performance computing
mRNA: Messenger RNA
QC: Quality control
RNA: Ribonucleic acid
RNA-Seq: RNA sequencing
