# Supplementary material for: Curare and GenExVis: a versatile toolkit for analyzing and visualizing RNA-Seq data
Source: BMC Bioinformatics. 2024 Mar 29;25:138. doi: 10.1186/s12859-024-05761-2 (PMC10979593; doi:10.1186/s12859-024-05761-2)
Supplement: Supplementary file 3 — Additional file 3: Supplementary figures and tables. [file 12859_2024_5761_MOESM3_ESM.docx]

# Figures, tables, and additional files

**Table S1: Tools and workflows for RNA-Seq analyses.**

| Name | Description | Latest updates | URL |
| --- | --- | --- | --- |
| Viper | - Snakemake - Workflow with customization via parameters - DGE analysis (starting with mapping of reads) for eukaryotes and prokaryotes | 2023 | https://bmcbioinformatics.biomedcentral.com/articles/10.1186/s12859-018-2139-9 |
| R-peridot | - Modularized DGE analysis with R (starting with gene expression abundance table) - Uses a graphical user interface (GUI) - Supports Microsoft Windows | 2019 | https://github.com/pentalpha/r-peridot |
| hppRNA | - Snakemake - RNA-Seq workflow system with multiple mapping/DGE variants - Usable for human and mouse | 2018 | https://academic.oup.com/bib/article/19/4/622/2918128 |
| TRAPLINE | - Galaxy workflow - Standardized pipeline for RNA-Seq data | 2021 | <https://bmcbioinformatics.biomedcentral.com/articles/10.1186/s12859-015-0873-9> |
| nf-core: rnaseq & differential-abundance | - Nextflow - 2 RNA-Seq workflows with alternative tool options - rnaseq: from raw sequencing reads until gene expression abundance table - differentialabundance: from abundance table until DESeq2 analysis - Online executable via Nextflow Tower | 2023 | https://nf-co.re/rnaseq  https://nf-co.re/differentialabundance |
| Galaxy | - Online platform for data analysis - Public server and hostable local server - Fully customizable DGE analysis via a large collection of available tools - Requires data upload to external server | 2023 | https://galaxyproject.org/ |

**Table S2: Websites, tools, and libraries for visualizing differential gene expression data.**

| Name | Type | Description | Latest updates | URL |
| --- | --- | --- | --- | --- |
| DrEdGE | Local web server | - Web server build with npm - Visualizations to transcript abundance tables and differential expression tables | 2020 | https://academic.oup.com/bioinformatics/article/36/8/2581/5695703 |
| ViDGER | R package | - Collection of R functions - Various visualizations for DGE data | 2021 | https://bioconductor.org/packages/release/bioc/html/vidger.html |
| DEBrowser | R web server | - R Shiny application - Various visualizations and analyses of DESeq2, edgeR, and limma results - Will do the differential analysis itself with DESeq2, edgeR, or limma | 2023 | https://bmcgenomics.biomedcentral.com/articles/10.1186/s12864-018-5362-x |
| DGEReport | R package | - Generates a report with various visualizations of DESeq2 and edgeR results | 2022 | https://bioconductor.org/packages/release/bioc/html/DEGreport.html |
| PIVOT | R web server | - R Shiny application - Numerous visualizations and analyses of DGE data - Will do the differential analysis itself with DESeq2 - Supports single-cell experiments | 2020 | https://bmcbioinformatics.biomedcentral.com/articles/10.1186/s12859-017-1994-0 |
| Degust | Web site and web server | - Web server and HTML report mode - Various visualizations and tables for DGE data - Can do the differential analysis itself with edgeR and limma in webserver mode | 2022 | https://degust.erc.monash.edu/ |


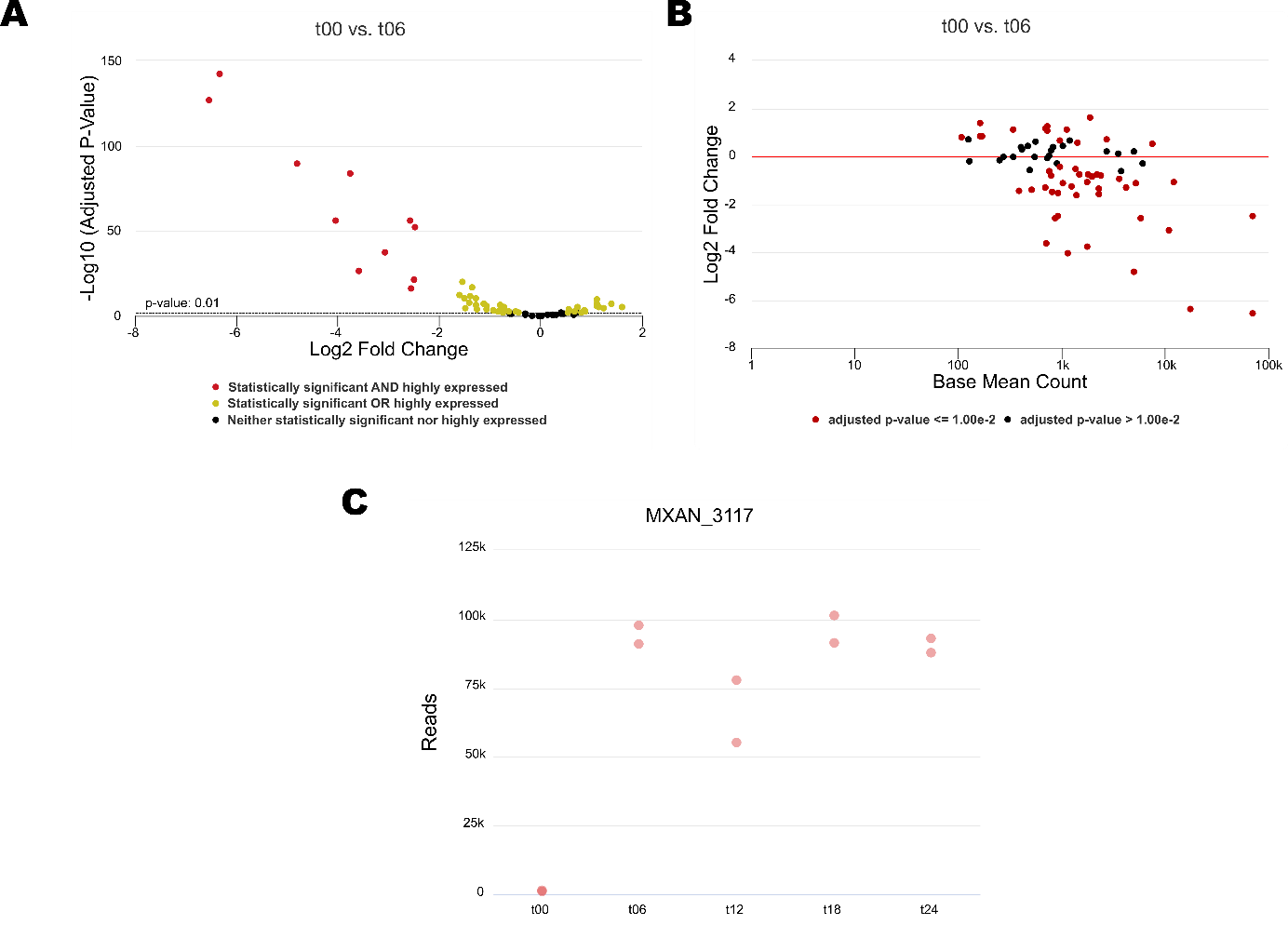


**Figure S1: Visualization examples of GenExVis.** The comparison 0h (t0) versus 6h (t6) was used for all visualizations. All plots are zoomable, and each point in the plot shows information about gene name, gene expression, and/or DESeq2 results by mouse over. In A and B, each point represents a single gene. In C, each point represents one sequencing sample. **A:** Volcano plot with log2(fold change) on the x-axis and -log10(adj. p-value) on the y-axis. **B:** MA plot with DESeq2 base mean count on the x-axis and log2(fold change) on the y-axis. C: Plot (gene MXAN_3117) from the “Top 10” feature in GenExVis sorted by log2(fold change)-increase between 0h and 6h with expression counts on the y-axis.

# Additional Files

File name: Additional file 1
File format: Excel spreadsheet (XLSX)
Title of data: Curare DGE results t0 vs rest
Description: Results of the Curare DGE analysis for every comparison with t0

File name: Additional file 2
File format: Comma-separated values (CSV)
Title of data: GenExVis subset export of t0 vs t6
Description: GenExVis export of the 72 CDS subset for the comparison t0 vs t6
